# Performance of a smartphone-based malaria screener in detecting malaria in people living with Sickle cell disease

**DOI:** 10.1371/journal.pdig.0000884

**Published:** 2025-06-09

**Authors:** Deborah Nimako Sarpong Obeng, Samuel Osei, Nii Kpakpo Brown, David Nana Adjei, Linda Eva Amoah, Ewurama Dedea Ampadu Owusu

**Affiliations:** 1 Department of Medical Laboratory Sciences, School of Biomedical and Allied Health Sciences, College of Health Sciences, University of Ghana, Legon, Accra, Ghana; 2 Department of Biology, University of Texas at Arlington, Arlington, Texas, United States of America; 3 Central Laboratory, Korle-bu Teaching Hospital, Korle-bu, Accra, Ghana; 4 Department of Immunology, Noguchi Memorial Institute of Medical Research, University of Ghana, Legon, Accra, Ghana; University of Washington, UNITED STATES OF AMERICA

## Abstract

Novel automated digital malaria diagnostic tests are being developed with the advancement of diagnostic tools. Whilst these tools are being evaluated and implemented in the general population, there is the need to focus on special populations such as individuals with Sickle Cell Disease (SCD) who have altered red blood cell morphology and atypical immune responses, which can obscure parasite detection. This study aimed to evaluate the diagnostic performance of one of such tools, the National Library of Medicine (NLM) malaria screener app in people living with sickle cell disease in a malaria-endemic country, Ghana. A descriptive cross-sectional study was conducted among SCD patients attending the Sickle Cell Clinic at Korle Bu Teaching Hospital in Accra, Ghana. Following informed consent, whole blood samples were collected and analyzed using the NLM malaria screener app, conventional microscopy, RDT, and Polymerase Chain Reaction (PCR), with PCR as the reference standard. The sensitivity, specificity, positive predictive value (PPV), and negative predictive value (NPV) of each diagnostic method were compared against PCR results. The NLM app identified the highest number of positive malaria cases, with 110 positive cases (36.2%), while both RDT and microscopy reported the highest number of negatives, with 287 negative cases (94.4%). Compared to PCR, the NLM app demonstrated a sensitivity of 89.5% and a specificity of 67.4%. RDT and microscopy displayed the same sensitivity as the NLM app, each achieving 89.5%. However, while RDT and microscopy had a specificity of 100%, the NLM app had a considerably lower specificity of 67.4%.The NLM malaria screener app shows promise as a preliminary screening tool for malaria in individuals with SCD. However, its lower specificity indicates a need for confirmatory testing to avoid potential overdiagnosis and mismanagement. Enhancements in the app’s specificity could further support its utility in rapid and accessible malaria diagnosis for people with SCD, aiding in timely management and treatment.

## Author summary

Malaria and sickle cell disease (SCD) are co-located in tropic regions globally. This can bring about interactions in the diagnosis, treatment and management of both diseases. Early detection of malaria parasites with accurate diagnostic tests can prevent severe crises and death in people living with SCD. The National Library of Medicine (NLM) malaria screener app is a new smart phone-based tool that is affordable and available for use in low resource areas and can be downloaded from the app store. We evaluated its performance among SCD patients at Korle Bu Teaching Hospital in Ghana. Our study compared results from the NLM app with other diagnostic tests, specifically, rapid diagnostic tests (RDT), microscopy, and Polymerase Chain Reaction (PCR), using PCR as the reference standard. The NLM app’s sensitivity was comparable to RDT and microscopy, however, its specificity was lower than the other methods. While the app identified the highest number of positive malaria cases (36.2%), its lower specificity suggests a need for confirmatory testing to prevent overdiagnosis. Our findings highlight the potential of digital malaria diagnostic tools for rapid screening in vulnerable populations such as people with SCD, with necessary refinements to improve accuracy.

## Introduction

Sickle cell disease (SCD) is known to be a group of genetic disorders marked by mutations in the gene encoding the haemoglobin subunit beta (HBB) where the amino acid valine is substituted for glutamic acid in the β-globin chain. It includes HbSS, HbSC, and HbS-thalassaemia [1]. It is estimated that more than 300,000 children are born each year with SCD throughout the world, and by 2050, that number is expected to rise to 400,000 [2]. Notably, up to 70% of these births take place in sub-Saharan Africa, where most of the infants who are affected die on an annual basis [3]. In Ghana, evidence suggests that each year, approximately 2% of newborns are diagnosed with SCD [4] whilst some areas have recorded prevalence as high as 16% [5,6].

Malaria shares a geographic location with SCD [7,8] and therefore interactions in terms of disease progression, diagnostics, treatment, and management between the two diseases have been documented [8–10]. Mortality from SCD is high among undiagnosed infants in sub-Saharan Africa, often due to infections like malaria [11–13]. While carrying only one sickle haemoglobin gene (HbS) can protect against malaria, children with HbSS, HbSC, and HbS-thalassaemia frequently suffer severe malaria-related complications [9].

Malaria can cause a pain or sequestration crisis in a patient with SCD and worsen the anaemia caused by sickle cell anaemia to the point where it becomes life-threatening [9]. Hence protecting SCD patients from malaria may help to lessen the crisis and all associated issues [14]. Due to the life-threatening consequence of severe malaria, such as cerebral malaria, severe anaemia, respiratory distress, and acute renal failure, severe malaria is a significant factor in the early mortality of sickle cell disease (SCD) patients in Africa [14,15].

Diagnosis of malaria infection in sickle cell patients is therefore critical for effective management of the disease, as early detection and treatment can prevent severe complications and death. One of the commonly used diagnostic methods for malaria is the microscopic examination of blood films. However, it is time-consuming, and the accuracy of the results is greatly influenced by the microscopist’s level of expertise [16]. In resource-limited areas, where most persons with SCD live, there are few trained medical professionals available for microscopic examination of blood smears [17]. To improve the performance of malaria parasite classification, many researchers have proposed automated malaria detection devices using digital image analysis which automates both picture acquisition and image processing [18]. Yu et al developed a smartphone-based malaria screener as an evidence-based tool to aid in the diagnosis of malaria, particularly in low-resource settings where access to microscopy or other diagnostic tests may be limited [19]. It can be downloaded from Google Play Store or Apple Store on cell phones. The slide screening procedure of the NLM Malaria Screener involves image capture, smear image analysis, result display, and the database, making data accessibility seamless [20]. The mobile application screens both thin and thick blood smear images for *Plasmodium falciparum* and other malaria species using high-resolution cameras and the processing capabilities of contemporary smartphones [20]. Throughout the screening process, the NLM Screener streamlines, standardizes, and lessens the need for human expertise [20].

With the advancement of malaria diagnostic tests and the development of automated and digital microscopy for malaria detection, there is a need for data on their performance in different malaria geographic settings and within different vulnerable populations. This study therefore aimed to assess the performance of the malaria screener developed by NLM for diagnosing malaria among people living with sickle cell disease.

## Materials and methods

### Study site and population

The Ghana Institute of Clinical Genomics (Sickle Cell Clinic) at the Korle Bu Teaching Hospital, a major referral Centre, sees an average of 50 sickle cell patients daily and has over 27,000 registered patients. This is done through an outpatient care department. When specialist care is required, patients are referred to the various units of the Korle Bu Teaching Hospital.

### Study design and participant selection

In this prospective cross-sectional study, sickle cell patients attending the sickle cell clinic during the study enrolment period of June - July 2023 suspected of having malaria and willing to consent, were enrolled. Ethics approval was obtained from the Ethical and Protocol Review Committee of the School of Biomedical and Allied Health Sciences (SBAHS), College of Health Sciences, University of Ghana - SBAHS/AA/MLAB/1O846918/2022–2023. Upon providing written informed consent demographic details such as age, sex, residence, and diagnostic history were recorded.

### Sample collection

Blood was collected using either capillary puncture or venipuncture, depending on the routine laboratory request made by the doctor on duty. For capillary puncture, the middle finger was pricked using a sterile lancet after disinfection with a 70% alcohol swab, yielding approximately 1ml of blood. Utilizing the venipuncture technique, the antecubital fossa was disinfected with a 70% alcohol swab, and 3 ml of blood was drawn using a needle. A quantity of this blood was also used for other routine laboratory examinations done at each patient visit to the sickle cell clinic. The collected blood was placed into ethylene diamine tetra-acetic acid (EDTA) tubes. A peripheral blood smear was prepared by creating a thick film from a drop of blood, which was stained with Giemsa stain for 15 minutes using a working solution prepared by mixing 1 part of Giemsa stock solution with 9 parts water [21]. The stained smears were then rinsed with water, air-dried, and examined microscopically by a trained microscopist using the x100 oil immersion objective [22]. Additionally, 100μl of blood was pipetted onto filter paper (Whatman No. 3) to create dried blood spots (DBS), with each spot containing 50μl of blood. These DBS samples were used for PCR-based malaria detection.

### Rapid Diagnostic Test (RDT)

About 5 µl of finger-prick capillary blood samples were applied on the sample pad on the test cassette (Bioline Malaria Ag P.f), and 4 drops of buffer were added (according to manufacturer instructions). The cassette was left to stand for 15 minutes before the results were read. Two bands showing on the “control” and “*P. falciparum*” area, meant the patient was malaria-positive and one band showing at the “control” area, meant the patient was malaria-negative.

### Blood film examination using Malaria Screener APP

**Fig 1:**
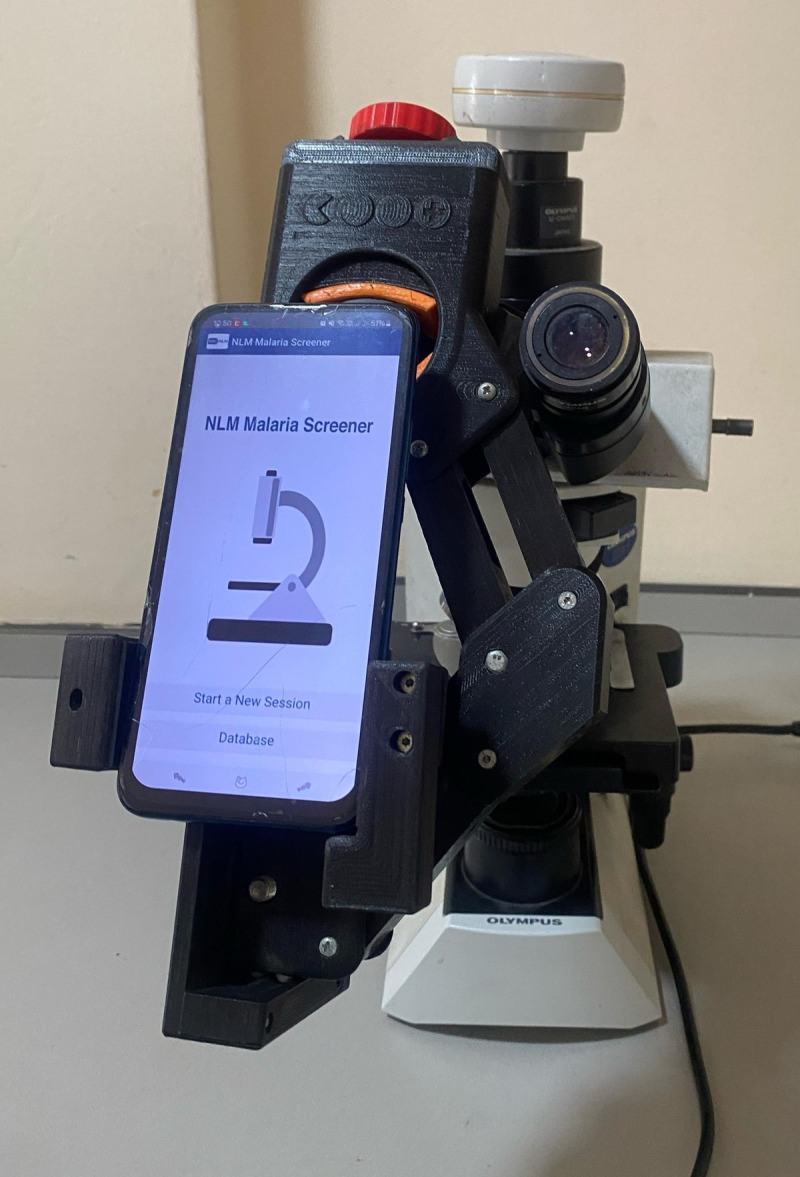
Setup - the downloaded NLM malaria screener app with the phone affixed to a microscope.

The NLM Screener mobile application was downloaded from Play Store with a Samsung Galaxy Note 8 and was launched on the phone. The prepared blood smear slide was placed on the stage of the microscope. The focus and magnification of the microscope were adjusted to the appropriate levels. The smartphone camera lens was kept close to the microscope eyepiece and aligned with the eyepiece to take a picture of the slide. Fig 1 is an image of the setup used in this study. The calibration tool of the camera was used to ensure the shot was aligned using the app’s default settings, which initially assumes sensitivity and specificity of 50% for analysis. Contrast, brightness, and other settings were modified to improve the image quality. While the blood smears were being screened, a medical laboratory scientist with over 6 years of working experience looked for suitable Field of Views (FoVs) for the app to capture and the app instantly processed the image on the phone. Certain areas of interest were marked in the image using the app’s annotation features. These two actions were repeated until a user-specified white blood cell (WBC) threshold of 200 was met (the threshold is 200 by default and can be changed by the user). The entire procedure was done according to developer instructions. The diagnostic findings and the results were then recorded.

### DNA extraction and conventional polymerase chain reaction

Two full smaller circles of spotted blood were punched from the filter paper and placed in Eppendorf/ microcentrifuge tubes. To extract DNA from the blood sample, an extraction buffer (Phosphate buffered saline (PBS) supplemented with Tween) was added to each sample to lyse the RBCs, and the Standard Operating Procedure (SOP) for the extraction of DNA in the lab was duly followed. Both NEST 1 and NEST 2 were used for Conventional PCR, with NEST 1 amplifying the human DNA and NEST 2 amplifying the *P. falciparum* species [23]. Primers specific to the target parasite that is Pf1 and Pf2 were amplified, and the DNA was extracted by PCR. Four cycle steps of the thermocycler were set for the samples, following the manufacturer’s instructions. Gel electrophoresis was used to analyze the PCR results, and the data was recorded.

### Statistical analysis

Data obtained were cleaned, analyzed, and interpreted using Statistical Package for the Social Sciences (SPSS) version 27. Categorical variables were summarized as frequencies and percentages. Continuous variables were summarized as means and standard deviations. Sensitivity and specificity with corresponding 95% confidence intervals were computed for each of the diagnostic tools using PCR as the gold standard.

### Ethical considerations

Ethical approval was sought from the Ethical and Protocol Review Committee of the School of Biomedical and Allied Health Sciences (SBAHS), College of Health Sciences, University of Ghana, number ref: SBAHS/AA/MLAB/1O846918/2022–2023, and permission sought from the Director of Ghana Institute of Clinical Genetics (Sickle Cell Clinic. Written informed consent was obtained from all study subjects.

## Results

**Fig 2:**
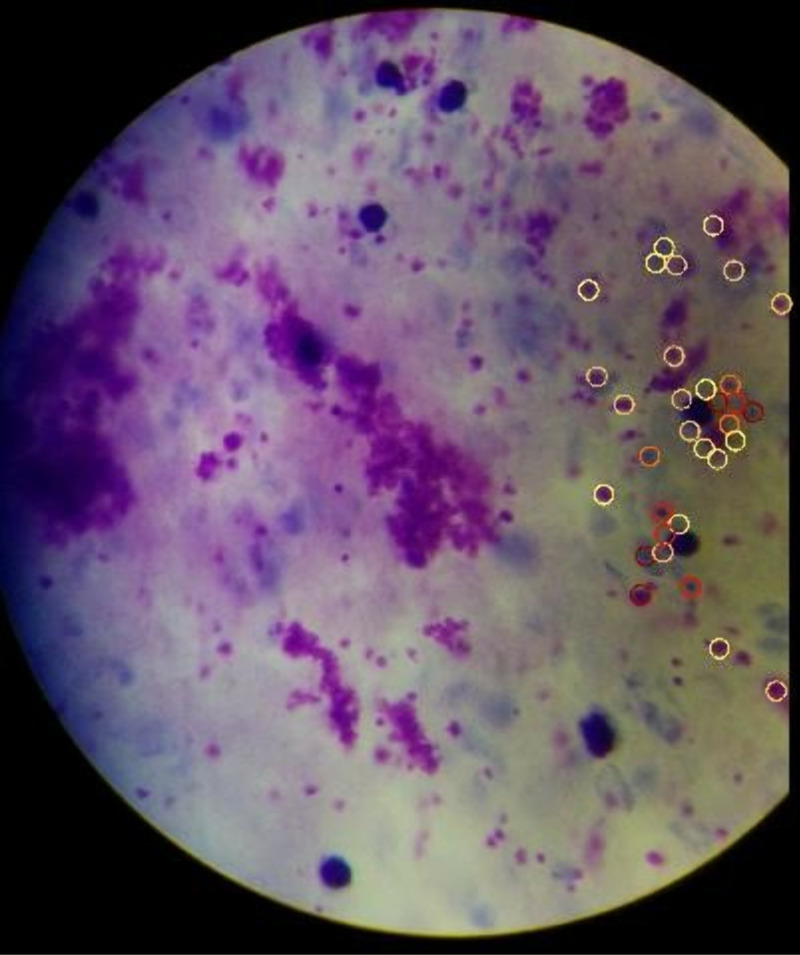
An image of infected malaria red blood cells (RBCs) examined using the NLM Malaria Screener App.

A total of 304 slides were prepared from the study participants, which culminated in 3040 images taken for thick blood smears (10 images/patient), indicated in Table 1. An example of the view of malaria-infected blood slide is in Fig 2. There were 98 (32.2%) males and 206 (67.8%) females, with age ranging from 11 to 72 years. The mean age of the study participants was 33.8 ± 13.4 years. The mean ages of males and females respectively, were 33.1 ± 13.0 years and 34.1 ± 13.6 years.

**Table 1:** Demographic Characteristics of Study Participants.

Variable	Frequency (n)	Percentage (%)
Sex	98	322
Males	206	67.8
Females		
Age group (years)	31	10.2
11-20	89	27.0
21-30	107	35.2
31-40	53	17.4
41-50	20	6.6
51-60	11	3.6
Over 60		
Recent antimalarial intake	10	3.3
< 3 months	25	8.2
3-6 months	269	88.5
No		
Sickling Status	250	82.2
Sickle cell SS (HbSS)	53	17.4
Sickle cell SS (HbSC)	1	0.4
S-beta-thalassemia (Hb S/β Th)		

The circles represent potential parasites present in the RBCs

### Sensitivity and specificity of diagnostic tests using PCR as reference method

#### NLM APP versus PCR.

The NLM App correctly identified 17 patients as positive for malaria (17 true positives) as shown in Table 2. The app incorrectly identified malaria (false positives) in 93 patients. Malaria parasites were correctly observed as being absent in 192 patients (true negatives), whilst indicating 2 false negatives. Based on the PCR reference standard, the NLM App seems to have a high sensitivity of 89.5% (95% CI: 75.6-100). It showed a lower specificity of 67.4% (95% CI: 61.9-72.8).

**Table 2:** Comparison of NLM App and PCR.

NLM App * PCR Cross tabulation				
		PCR		
		Negative n (%)	Positive n (%)	Total n (%)
NLM App	Negative	192 (67.4)	2 (10.5)	194 (63.8)
	Positive	93 (32.6)	17 (89.5)	110 (36.2)
Total Sensitivity = (89.5%) (95% CI:75.6-100) Specificity= (67.4%) (95% CI: 61.9-72.8)		285 (100.0)	19 (100.0)	304 (100.0)

CI – Confidence Interval; RDT -Rapid Diagnostic Test

#### Comparison of RDT and PCR.

The RDT accurately determined that 17 individuals had malaria (true positive) using PCR as the reference standard (Table 3). However, there were no false positives detected. Correctly identified malaria-negative patients (true negative) were 285, whilst incorrectly identified ones (false negatives) were 2. Here sensitivity and specificity of the RDT were 89.5% (95% CI: 75.7-100) and 100% (95% CI: 100–100) respectively.

**Table 3:** Comparison of RDT and PCR.

		PCR		Total n (%)
		Negative n (%)	Positive n (%)	
RDT	Negative	285(100.0)	2(10.5)	287(94.4)
	Positive	0(0.0)	17(89.5)	17(5.6)
Total Sensitivity = (89.5%) (95% CI: 75.7-100) Specificity = (100%) (95% CI: 100–100)		285(100.0)	19(100.0)	304(100.0)

CI – Confidence Interval; RDT -Rapid Diagnostic Test

#### Microscopy versus PCR.

Using PCR as the reference standard, microscopy accurately identified individuals with malaria (true positive) in 17 patients (Tables 4 and 5). However, there were no false positives. True negatives totalled 285, whilst false negatives were only 2.

**Table 4:** Comparison of Microscopy and PCR.

Microscopy * PCR Cross tabulation				
		PCR		Total n (%)
		Negative n (%)	Positive n (%)	
Microscopy	Negative	285 (100.0)	2 (10.5)	287 (94.4)
	Positive	0 (0.0)	17 (89.)	17 (5.6)
Total Sensitivity = (89.5%) (95% CI: 75.7-100) Specificity = (100%) (95% CI: 100–100)		285 (100.0)	19 (100.0)	304 (100.0)

CI – Confidence Interval

**Table 5:** A table showing results for true positives, true negatives, false positives, and false negatives of the NLM APP, RDT, and Microscopy with PCR as reference standard.

Reference Standard	Target Species	Method	All cases	RS +	RS −	TP	FP	TN	FN	Accuracy (95% CI|)	Sensitivity (95% CI)	Specificity (95% CI
PCR	*Plasmodium* *falciparum*	NLM	304	19	285	17	93	192	2	68.8% (63.4-74.1)	89.5% (75.6-100)	67.4% (61.9-72.8)
		RDT	304	19	285	17	0	285	2	99.3% (98.4-100)	89.5% (75.7-100)	100% (100-100)
		Microscopy	304	19	285	17	0	285	2	99.3% (98.4-100)	89.5% (75.7-100)	100% (100-100)

## Discussion

This study demonstrates the sensitivity and specificity of the NLM app was further from the PCR reference, unlike conventional microscopy and RDT results. Overall, sensitivity was high in the NLM app, comparable to the RDT and microscopy tests; however, specificity for NLM was markedly lower in the same population.

A diagnostic tool with good sensitivity and specificity, that does not require much expertise and is efficient can be a good choice for early malaria detection in people with sickle cell disease. The low specificity of this app has previously been observed in a patient-level study in Sudan, and this was attributed to the presence of parasite-like staining artifacts that this particular model was unable to distinguish [20]. Additionally, smartphone camera variability in image quality and lighting conditions likely contributes to inconsistent app performance, impacting its diagnostic accuracy [20]. These technical limitations warrant further algorithm refinement and validation to enhance the app’s diagnostic accuracy, particularly in specialized populations. The app developers have indicated they are considering an improvement in the training set from more real-world data, amongst other things [20].

Furthermore, the high number of false-positive results in the NLM app in this study may be concerning, as they could lead to unnecessary antimalarial treatment. In recent years, an increased risk of drug resistance has been documented in Africa and there have been urgent calls to address them [24,25]. The widespread misuse of antimalarials in treating undiagnosed fevers and inaccurate diagnosis are major contributing factors [26]. Globally, more awareness of the contribution of antimalarial use to antimicrobial resistance (AMR) is being created as resistance spreads in Africa. Thus, concerted efforts at controlling the use of antimalarials, one of which is the reduction in unnecessary prescriptions from false positives, is imperative.

A concomitant unneeded antimalarial treatment from false positives in people with sickle cell disease could also impact other medications that they regularly use to manage the disease. However, in our study, as we did not conduct a case-control observation of the performance of the app and so did not determine correlation between FP and SCD. Nonetheless, false positives in any population could result in unnecessary administration of antimalarial drugs. In Ghana, hydroxyurea (HU) is a treatment for sickle cell disease that has been available since 2019 and in 2021 was included in the National Health Insurance list of provided medicines [4]. Additionally, the long-standing recommended treatment for uncomplicated malaria in Ghana, including people with sickle cell disease is Artemisinin-based Combination Therapy (ACT) which consists of Artesunate-Amodiaquine (AS-AQ), Artemether-Lumefantrine (A-L) or Dihydroartemisinin-Piperaquine (DHAP) [27]. However, research on the potential interactions between HU and ACT is limited. One recent study in Ghana observed differences in haematological and clinical chemical parameters in their study of sickle cell patients who had been diagnosed with uncomplicated malaria and were on hydroxyurea and antimalarial Artemether-Lumefantrin [28]. Whereas another *in vitro* study looking at the effects of HU action on the anti-parasite activity of antimalarials and its efficacy recommended its safety and long-term use [29]. Our study did not collect information on hydroxyurea medication from the patients, however, with the easily accessible HU treatment among the Ghanaian population, frequent exposure to antimalarials from wrongly diagnosed malaria could result in drug-drug interactions. This therefore requires extensive studies in larger cohorts of people with sickle cell disease.

There were some inherent limitations of this study that if addressed in further studies, can shed more light on the dynamics of this screening tool. For instance, even though the two false negative samples were the same for RDT, microscopy and NLM app, further PCR analysis of the parasitemia of the false negatives was not conducted in this study due to funding limitations and so the determination of the detection limit was not possible. Secondly, this study was not large enough and did not include data from a control group nor HU medication history to establish the wider representation of the performance of the app. Nonetheless, this research has provided additional baseline data on the performance of the screening app in a different population, people with sickle cell disease in Ghana, a malaria-endemic sub-Saharan country. In prior studies evaluating the NLM app, it demonstrated robust sensitivity but varied specificity across different populations [19,20]. Missed malaria cases can escalate to severe malaria and cerebral malaria and possible death in children especially [30] therefore the capacity of this app to reduce the chances of that occurring is advantageous.

Even though the low specificity of the app in our study highlights the need for additional research across diverse settings and patient demographics, especially among sickle cell populations, for whom no prior studies exist, it can serve as an accessible initial screening tool in remote areas where other diagnostic methods may be unavailable. The focus at this stage of diagnostic development may not necessarily be on replacing current confirmatory diagnostics like microscopy or PCR, particularly in populations with coexisting conditions that may complicate malaria diagnosis. As a relatively new technology, further studies are needed to assess the app’s performance across varied populations and geographic settings to understand and address its specific limitations [27].

Despite these limitations, our study provides baseline and novel information on the performance of the NLM app in people with sickle cell disease in Ghana, a sub-Saharan geographic location with perennial malaria transmission. While the NLM app shows promise as a cheap and accessible screening tool for malaria which does not require much expertise, its low specificity suggests that it could be used alongside confirmatory diagnostic methods, especially for vulnerable populations like those with sickle cell disease. Improving its algorithm to better handle haematologic variability and conducting more studies on diverse populations could enhance its utility and reliability.
